# Influences of nutrition and adiposity on bone mineral density in individuals with chronic spinal cord injury: A cross-sectional, observational study

**DOI:** 10.1016/j.bonr.2015.02.002

**Published:** 2015-02-18

**Authors:** Irena Doubelt, Julia Totosy de Zepetnek, Maureen J. MacDonald, Stephanie A. Atkinson

**Affiliations:** a Department of Pediatrics, McMaster University, 1280 Main St. West, Hamilton, ON L8S 4K1, Canada; b Department of Kinesiology, McMaster University, 1280 Main St. West, Hamilton, ON L8S 4K1, Canada

**Keywords:** AIS, American Spinal Injury Association Impairment Scale, DRI, dietary reference intakes, EAR, estimated average requirement, FFQ, food frequency questionnaire, IOM, Institute of Medicine, SCI, spinal cord injury, SLOP, sublesional osteoporosis, UPLC/MS–MS, ultra high performance liquid chromatography tandem mass spectrometry, VAT, visceral adipose tissue, WC, waist circumference., Spinal cord injury, Sublesional osteoporosis, Bone mineral density, Nutritional status, Adiposity

## Abstract

**Background:**

Dietary inadequacy and adiposity, both prevalent in the chronic spinal cord injury (SCI) population, are known to influence bone turnover and may be potential modifiable risk factors for the development of sublesional osteoporosis following SCI. This pilot study in an SCI cohort aimed to assess measures of nutrition and obesity, to determine if these measures were associated with bone mineral density (BMD), and to compare these measures to a non-SCI control cohort.

**Methods:**

In a cross-sectional observational study, volunteers with chronic SCI (> 1 year post-injury, lesions from C1 to T12 and severity category A–D by the American Spinal Injury Association Impairment Scale) were assessed, and 8 non-SCI individuals were recruited as a comparison group. BMD at the femoral neck (FN) and lumbar spine (LS), and an estimate of visceral adipose tissue (VAT) from lumbar vertebrae 1 through 4 were measured using dual energy X-ray absorptiometry (DXA); nutrient intake of calcium, vitamins D & K, and protein were estimated using a food frequency questionnaire; plasma 25-hydroxyvitamin D (25(OH)D) was analyzed using ultra-high performance liquid chromatography/tandem mass spectroscopy; and serum leptin, adiponectin and insulin were analyzed using a multiplex assay.

**Results:**

A total of 34 individuals with SCI (n = 22 tetraplegic; n = 12 paraplegic; 94% male) who averaged 12.7 (9.0) years post-injury, age 40.0 (10.9) years and % body fat of 28.4 (7.3) were assessed. Multiple linear regression analyses in the SCI cohort showed significant associations between BMD at the FN and LS with leptin (FN: r = 0.529, p = 0.005; LS: r = 0.392, p = 0.05), insulin (FN: r = 0.544, p = 0.003; LS: r = 0.388, p = 0.05), and VAT percent (FN: r = 0.444, p = 0.02; LS: r = 0.381, p = 0.05). Adiponectin was only correlated with LS BMD (r = 0.429, p = 0.03). No significant relationships were found between BMD and serum 25(OH)D, or intakes of calcium, vitamins D & K, and protein. Intake of vitamin D was adequate in 69% of participants with SCI, where 91% of those persons consumed either vitamin D and/or multivitamin supplements. Vitamin D status was similar between SCI and non-SCI groups as was sub-optimal status (25(OH)D < 75 nmol/L) (60% of SCI compared to 50% of non-SCI). Participants with SCI had significantly lower FN BMD in comparison to non-SCI controls (p = 0.001).

**Conclusions:**

Compromised BMD among individuals with SCI was not associated with a deficiency of vitamin D or other bone nutrients. The observed positive associations between BMD and leptin, insulin, adiponectin and VAT provide a framework to evaluate links between adiposity and bone health in a larger SCI cohort.

## Highlights

• In SCI subjects, BMD was positively associated with adiposity.
• Adipokine regulation may be more important for bone status in chronic SCI.
• Compromised BMD in SCI was not attributed to suboptimal vitamin D or bone nutrients.
• Widespread variability in SCI stage/severity may have influenced lack of association.
• Suboptimal 25(OH)D status was seen in 60% of an SCI cohort, despite vitamin D supplement use in 63%.

## 1 Introduction

Sublesional osteoporosis (SLOP) is a disease specific to the spinal cord injury (SCI) population associated with excess bone resorption paired with reduced bone formation below the level of lesion (Craven et al., 2009). The dramatic loss of bone mineral density (BMD) increases the risk of acquiring fragility fractures at the distal femur, proximal tibia, and hip regions (Craven et al., 2009). There is a preservation of BMD at the lumbar spine (LS) during the chronic phase of SCI, which may be due to the maintenance of load on the spine while sitting in a wheelchair (Biering-Sorensen et al., 1988). However the trabecular composition of the LS with high bone turnover can cause changes in BMD prior to fracture (Craven et al., 2009) and many vertebral compression fractures at this site go undetected in other clinical disorders such as steroid drug-induced bone loss (Rodd et al., 2012a). The occurrence of SLOP is estimated to be up to 82% in individuals with SCI (Craven et al., 2009). Although nutrition and adiposity have known roles in the regulation of bone metabolism, the importance of these factors compared to loss of weight bearing in those with SCI is not well defined.

Sub-optimal vitamin D status is common in the SCI population (Hummel et al., 2012, Bauman et al., 1995, Bauman et al., 2005, Oleson et al., 2010, Nemunaitis et al., 2010). It is attributed to limited exposure to sunlight due to reduced mobility, use of medications that accelerate vitamin D metabolism, and/or low vitamin D intake due to calcium-restricted diets during acute care to avoid hypercalciuria (Bauman et al., 1995). Intake of calcium may thus also be compromised. Whether persons with greater fracture risk, such as the SCI population, require nutrient intakes higher than the dietary reference intakes suggested for the average population is unknown (Hanley et al., 2010, WHO Scientific Group, 2003).

Other factors that may influence bone formation and resorption in persons with SCI include pro-inflammatory cytokines released from excess adipose tissue (Cao, 2011). Leptin has been suggested to regulate bone metabolism primarily through the peripheral pathway promoting bone formation (Turner et al., 2013, Khosla, 2002). Leptin receptors on osteoblasts increase osteoblast differentiation while decreasing adipocyte differentiation, as they are both derived from a common multi-potential mesenchymal stem cell lineage (Thomas, 2003). Excess adiposity also increases exogenous insulin, which binds to insulin receptors on osteoblast cells and directly induces osteoblast activity (Karsenty and Ferron, 2012). Similarly, adiponectin may be a biomarker for bone loss and fracture risk as it is inversely related to BMD and central adiposity (Liu et al., 2013). Alternative theories regarding the fat–bone relationship suggest that mechanical loading by fat and muscle mass on bone tissue is associated with increased osteoblast activity, strengthening bones in regions of high stress (Jiang et al., 2006). In persons with SCI, body weight percentage as adipose tissue mass can be 8–18% higher than in age-, height-, and/or weight-matched non-SCI controls (Buchholz and Bugaresti, 2005). Following SCI, adipose tissue accumulation in parallel with loss of muscle and bone tissue indicates a disruption in adipose-associated bone metabolism (Jiang et al., 2006).

The presence of SLOP (Craven et al., 2009), nutrient inadequacy (Walters et al., 2009), and obesity (Rajan et al., 2008) in the SCI population suggests that a unique cross-regulation may exist. Characterizing relationships between markers of these physiologic states will further our understanding of bone health in the SCI population and may help in the detection and treatment of SLOP. This pilot study sought to assess bone-related measures of nutrition and obesity in a SCI cohort and determine if these measures are associated with BMD at the femoral neck (FN) and LS. Secondary goals were to identify correlates of suboptimal vitamin D status (e.g. winter assessment, vitamin D intake), and compare measurement outcomes to a small representative sample of non-SCI individuals.

## 2 Methods

### 2.1 Study setting and population

The study was conducted at McMaster University in Hamilton, Ontario, Canada; data were collected between March 2011 and April 2013. The Hamilton Integrated Health Sciences Research Ethics Board approved the study protocol and consent was obtained from each volunteer. Individuals enrolled in the study were at least 12 months post-SCI (traumatic or non-traumatic) ranging in impairment from C1 to T12, and ranging in severity from A to D as categorized by the American Spinal Injury Association Impairment Scale (AIS) (Kirshblum et al., 2011). The non-SCI individuals serving as a comparison group were matched for sex, age, waist circumference (WC), and body mass index (BMI).

### 2.2 Outcome measures

#### 2.2.1 Medical history and demographics

Demographics, injury characteristics, and medical history were obtained by interview. Body mass was measured using a digital wheelchair scale (Detecto BRW-1000 Digital Bariatric Wheelchair Scale, DETECTO, Webb City, MO, USA) to the nearest 0.1 kg and body length was measured on the right side of the body while lying supine to the nearest 0.1 cm. WC was taken in the supine position after normal expiration immediately below the lowest rib with the same tape measure as used for participant length (Gulick II) (Edwards et al., 2008). For each WC measurement, the tape measure was placed directly on the skin with the participants' arms by their sides. Each measurement was taken to the nearest 0.1 cm.

#### 2.2.2 Body composition

Dual energy X-ray absorptiometry (DXA) scans were performed using Hologic QDR-4500A (Hologic Inc., Waltham, MA, USA). Scan acquisition and analyses were completed following the manufacturers' guidelines for assessing areal BMD (aBMD) at the LS (lumbar vertebrae 1–4 or a minimum of two consecutive vertebrae) and FN of the left hip (Rajan et al., 2008); body fat and visceral adipose tissue (VAT) were analyzed from a whole body scan, where a demarcated region of interest from lumbar vertebrae 1 through 4 quantified VAT (Glickman et al., 2004). Quality control tests were performed daily using a phantom, and measurements were maintained within the manufacturer's standards of < 1%. Both T and Z-scores were calculated using a reference database for non-SCI individuals provided by the Hologic software; T-scores were used for participants over 50 years and Z-scores were used for participants under 50 years (Schousboe et al., 2013).

#### 2.2.3 Food and supplemental nutrient intake

Average daily dietary intakes of calcium, vitamins D and K, and protein were assessed using a previously validated food frequency questionnaire (FFQ) (Pritchard et al., 2010). Supplement use of multivitamins, calcium, and vitamin D were documented regarding amount(s) and duration of use. Dietary nutrient intake refers to the consumption of the nutrient from food, whereas absolute nutrient intake refers to the consumption of food plus supplements. To assess the adequacy of nutrient intake, reported intakes were compared to the estimated average requirement (EAR) for vitamin D (400 IU/day), calcium (800 mg/day) and protein (0.66 g/kg/day) and the adequate intake of vitamin K (120 μg/day) for adults aged over 18 years as recommended by Health Canada (Institute of Medicine, 2001, Institute of Medicine, 2011, Institute of Medicine, 2002).

#### 2.2.4 Blood collection and analyses

Blood samples collected following a 12-hour fast were spun and frozen at − 80 °C. Plasma 25(OH)D was quantified in triplicate using ultra high performance liquid chromatography tandem mass spectrometry (UPLC/MS–MS). The protocol for sample preparation was based on the Waters Alliance and Hymoller and Jensen protocol for HPLC LC/MS–MS with minor modifications (Hymoller and Jensen, 2011, Calton et al., 2008). The coefficient of variation (CV) for low and high 25(OH)D quality control measurements run five times was < 10%. Although there is no universal definition for optimal 25(OH)D status for bone health, the Institute of Medicine (IOM) defines adequacy as > 50 nmol/L (Institute of Medicine, 2011). We defined sub-optimal vitamin D status as < 75 nmol/L as recommended by the International Symposium on the Nutrition Aspects of Osteoporosis for persons with increased risk of fracture (Dawson-Hughes et al., 2005). Serum samples were analyzed for leptin, insulin, and adiponectin using the Milliplex® Map Kit for Human Adipokine Magnetic Bead Panel 2 (Millipore Corporation, Billerica, CA) in duplicate.

### 2.3 Statistical analyses

Descriptive statistics were computed by calculating the mean (SD) and count (percent) for categorical variables. The primary objective was explored using multiple linear regression analyses to associate BMD (LS and FN) with calcium intake, 25(OH)D status, leptin, insulin, adiponectin, and VAT. To address the secondary objective of characterizing vitamin D status among those with SCI, the sample was dichotomized based on optimal (≥ 75 nmol/L) and suboptimal (< 75 nmol/L) 25(OH)D. Univariate logistic regression analyses were then performed to identify whether the following characteristics were associated with suboptimal vitamin D status: winter assessment, inadequate absolute vitamin D intake (as defined by EAR), no vitamin D supplement use, no multivitamin supplement use, and medications affecting vitamin D metabolism. Seasonal variation of 25(OH)D was defined by samples collected in winter (November to April) or summer (May to October) months. Odd ratios (ORs), 95% confidence intervals (CI) and p-values were reported. Independent sample *t*-tests were performed between the whole SCI group (n = 34) and the SCI (n = 8) to ensure that our matched sample was representative of our cohort and between SCI (n = 8) and the matched non-SCI comparison group (n = 8), where p-value < 0.05 was considered significant. Statistical analyses were performed using the Statistical Program for Social Sciences (SPSS) (version 19.0; SPSS Inc., Chicago, IL).

## 3 Results

### 3.1 Recruitment and sample size

A total of 34 SCI and 8 non-SCI participants were recruited to participate in the study, with a complete data set for 27 SCI and 8 non-SCI participants. Reasons for missing data included: metal interference with DXA, patient's weight exceeded the maximum capability of the DXA machine, loss of communication, and one participant declining blood draw.

### 3.2 Participant characteristics

Participant and injury characteristics of the cohort consisting of 34 wheelchair-bound Caucasian participants with chronic SCI are summarized in Table 1. Neurological lesions varied from C1 to T11 and severity category from A to D (A = 13, B = 4, C = 16, D = 1) by AIS. Sixty-two percent of the male participants with SCI were obese when considering their body fat percent (> 25%), and 100% of the female SCI participants were obese (> 32%) (WHO Consultation, 2000). The 8 male non-SCI participants who were sex, age, WC- and BMI-matched to 8 of the participants with paraplegia had a mean age of 42.5 (6.7) years, BMI of 25.8 (3.8) kg/m^2^, WC of 91.9 (9.48) cm and 20.70% body fat.

### 3.3 Outcome measures

#### 3.3.1 Bone mineral density and visceral adipose tissue

BMD and VAT outcomes are summarized in Table 2. Based on the FN T and Z-scores, 32% of participants with SCI had lower than expected Z-scores for age and sex (≤ − 2), 11% were at an increased risk of fracture with low bone mass (T-score − 1 to − 2.5), 4% were at high risk of fracture and would be diagnosed with osteoporosis (T-score ≤ − 2.5) (Schousboe et al., 2013). SCI compared to non-SCI participants had a significantly lower FN BMD (p = 0.001) (Table 2). All other measurement outcomes were not significantly different between SCI and non-SCI.

#### 3.3.2 Dietary and supplemental nutrient intake

Intakes of vitamin D, calcium, vitamin K, and protein are summarized in Table 2. Vitamin D supplements were consumed by 63% of the SCI cohort in amounts ranging from 500 to 2000 IU per day, with one individual reporting taking 50,000 IU weekly at the time of assessment; 50% of participants were consuming at least 1000 IU per day not including a multivitamin. SCI compared to non-SCI participants had a significantly higher vitamin D intake Table 2. Calcium supplements of 300 to 2200 mg/day were consumed by 41% of participants with SCI and multivitamins containing between 400 and 800 IU of vitamin D and between 200 and 500 mg of calcium by 69% of participants. Without taking into account supplement intake (i.e. obtained from food only), 13% of the cohort fell below the EAR for calcium and 84% for vitamin D. Mean vitamin K and protein intakes were similar across SCI and non-SCI groups and exceeded the adequate intake of vitamin K (90–120 μg/day) and EAR of protein (0.66 g/kg/day) for females and males over 18 years (Table 2).

#### 3.3.3 Vitamin D status

Suboptimal plasma 25(OH)D status (< 75 nmol/L) was observed in 60% of the SCI cohort, of which 10% were truly deficient (< 30 nmol/L). Mean (SD) plasma 25(OH)D in the optimal category was 92.3 (12.0) nmol/L (n = 13) while in the suboptimal category was 54.4 (14.9) nmol/L (n = 20) for participants with SCI. Univariate logistic regression analyses revealed that participants with SCI not taking multivitamin supplements had increased odds of having suboptimal vitamin D status (OR = 0.171, CI = 0.033–0.893, p = 0.036). The mean 25(OH)D values for individuals with SCI consuming multivitamin supplements was 86.9 (21.2) nmol/L (n = 10) and for those without supplements was 63.1 (20.4) nmol/L (n = 23). No other potential correlates of deficient vitamin D status were significant in the SCI cohort. The vitamin D status of the SCI and non-SCI comparison groups was not significantly different, and 50% of the non-SCI group had suboptimal plasma 25(OH)D status (Table 2).

#### 3.3.4 Bone and adiposity measures

**Fig. 1:**
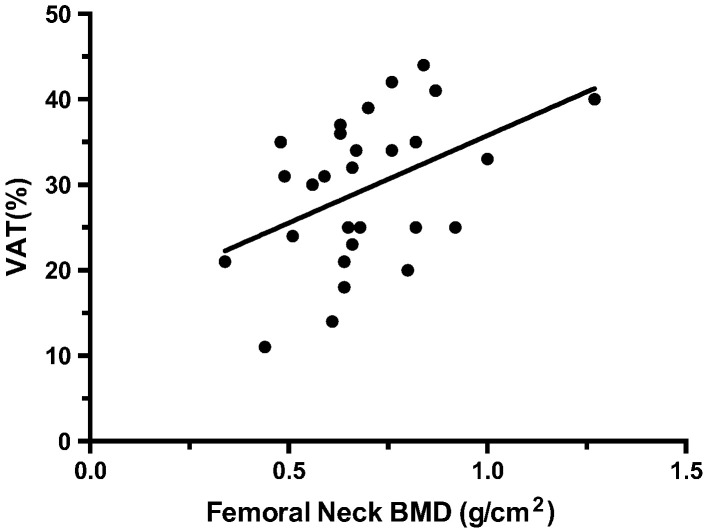
Relationship between bone mineral density (BMD) at the femoral neck (g/cm^2^) and visceral adipose tissue (VAT) (%) (r^2^ = 0.444; p-value = 0.018).

In participants with SCI, BMD at the FN and LS was significantly associated with leptin, insulin and VAT%, as was LS BMD with adiponectin (Table 3). An example of the significant positive, moderate correlation found between FN BMD and VAT is presented in Fig. 1. None of the other outcome measures were significantly related to BMD at the FN or LS.

## 4 Discussion

In our study cohort of 34 adults with chronic SCI, BMD at the FN and LS were associated with circulating adipokines and VAT, but not with any measure of nutrition. Sub-optimal bone status at the FN was significantly greater in participants with SCI than in a matched healthy reference group of non-SCI persons.

The positive associations found between BMD and VAT, leptin, insulin and adiponectin in the present study can be attributed to several potential mechanisms (Qin et al., 2010). It is postulated that higher body weight increases mechanical loading on bones, activating osteoblast cells for bone formation (Qin et al., 2010). Alternatively, metabolic factors may have an impact on BMD; greater adipose tissue is associated with higher secretion of bone-active hormones such as leptin and insulin, thereby directly stimulating osteoblast activity (Qin et al., 2010). Since the subpopulation of individuals with SCI experience a lack of mechanical loading below the level of lesion, metabolic influences from systemic hormone regulation are likely major contributors to BMD regulation.

Associations between adiposity measures and BMD were previously reported in a chronic SCI cohort (Doherty et al., 2014, Bauman et al., 2006); our data support and extend the proposal that adipokines may be candidate biomarkers for BMD in SCI-related sublesional osteoporosis. A previous study reported an inverse relationship between circulating adiponectin and SLOP in SCI wheelchair users rather than walkers, but the site of BMD measure was not indicated (Doherty et al., 2014). These findings suggest that osteoprotective benefits of obesity require mechanical loading in order to mitigate the resorptive effects of adiponectin (Karsenty and Ferron, 2012). Another study compared individuals with SCI to their non-SCI monozygotic twin, and reported significant positive relationships between leg BMD or BMC with total body fat percent, leg fat mass and serum estradiol in the SCI group, where only leg lean mass was correlated to leg BMD or BMC among the non-SCI twins (Bauman et al., 2006). The markers of adiposity in relation to lower extremity BMD are consistent with our findings. This relationship may be site-specific due to the redistribution of fat and muscle mass following SCI, where leg fat mass was the most significant predictor of leg BMD/BMC (Bauman et al., 2006). The positive association between adiponectin and BMD at the LS, but not the FN site found in our study, could be specific to the site of bone preservation due to the constant weight bearing in a seated position. LS preservation is evident in the comparable LS BMD of non-SCI controls. Alternatively, DXA has been found to overestimate the LS BMD in SCI due to confounding osteophytes or heterotrophic ossification (Bauman et al., 2009), and may mask bone loss following injury.

Following SCI, the gain in adipose tissue concurrent with the loss of lean and bone tissue results in a redistribution of body composition and a decreased metabolic rate (Buchholz et al., 2003). Since people with SCI have higher adipose mass, lower circulating adiponectin compared to non-SCI controls would be expected. Our results found slightly higher circulating adiponectin in SCI than non-SCI as seen in previous studies (Edwards et al., 2008, Wang et al., 2005), indicating that the hormonal mechanism of adiponectin on fat and bone metabolism may differ in persons with SCI. Alternatively, low resting metabolism after SCI may protect against hypoadiponectinemia (Ruige et al., 2005). A further explanation for the unexpected adiponectin findings is that persons with SCI produce biologically inactive forms of adiponectin (Sato et al., 2001, Scherer et al., 1995). The analysis methodology used for assessing circulating adiponectin measures various forms including both biologically active and inactive adiponectin, possibly impacting the results (Wang et al., 2005).

Although nutritional status was not associated with bone outcomes in the SCI cohort, it is worth noting that to achieve recommended intake of nutrients, the participants with SCI were highly reliant on supplements; 73% of the participants reported calcium, vitamin D and/or multivitamin supplementation. Vitamin D was the main nutrient with suboptimal intakes when accounted from food sources only. Frequent use of nutrient supplementation has been observed previously in a cohort of 77 adults with SCI, where 50% of participants consistently took multivitamin, calcium and vitamin D supplements (Opperman et al., 2010). Quite remarkably, despite the observed frequency of vitamin D supplementation, 60% of the participants with SCI demonstrated suboptimal 25(OH)D status. These findings align with previous reports where 32% of persons with SCI (n = 100) had suboptimal 25(OH)D status (defined as < 40 nmol/L) (Bauman et al., 1995). However, when the cut-off of 70–80 nmol/L is employed, more recent reports of suboptimal 25(OH)D status were as high as 93% in SCI cohorts (Wong et al., 2014). Multivitamin use was the only correlate associated with vitamin D inadequacy, most likely due to the high amounts of calcium and vitamin D present and other nutrients improving their absorption. Taken together, data from the present study and other reports indicate that greater intakes of vitamin D with more reliance on supplementation as well as clinical monitoring may be necessary for reaching adequate 25(OH)D status in SCI populations.

The lack of a relationship between BMD and skeletal nutrient intake or 25(OH)D status may be attributable to the wide variability of injury characteristics in our cohort, or other factors known to influence low BMD including: injury duration greater than 10 years, motor complete injury, female gender, BMI less than 19, medications that increase vitamin D metabolism and excessive cigarette smoking or alcohol intake (Hummel et al., 2012). The lack of impact of nutrient intake on bone status was observed in a study examining the factors contributing to steroid-induced osteoporosis in children with rheumatic disorders or nephrosis, possibly due to the overwhelming impact of glucocorticoid use (Huber et al., 2010, Feber et al., 2012, Rodd et al., 2012b). Significant associations between 25(OH)D status and BMD at the hip have been observed in non-SCI women over the age of 60 (Dawson-Hughes et al., 2005), but this relationship has not been previously reported in SCI. In our SCI cohort, bone loss appeared site specific at the FN, whereas bone mass was preserved in the LS. Future research should include evaluations of BMD at the proximal tibia and distal femur sites, where the highest incidence of fractures occurs (Craven et al., 2009). The observation of significantly lower FN BMD in our participants with SCI in comparison to non-SCI was expected since aBMD is known to decline by 20–32% within the first year of injury, particularly in the lower extremities (Craven et al., 2009).

Limitations to the current study include the cross-sectional observational design, which does not allow for observations to be made with regards to the chronology of outcomes or causality of relationships between different outcomes. Many of our participants were recruited from the MacWheelers Rehabilitation Centre at McMaster University and may not be generalizable to all persons with SCI due to their habitual exercise routine. In addition, the small and heterogeneous sample size did not allow confounding covariates of inadequate 25(OH)D status and sub-optimal bone status to be controlled.

## 5 Conclusions

The results of this study found that markers of adiposity were closely related to BMD at the FN and LS skeletal sites in adults with chronic SCI, while nutrition and 25(OH)D status showed no association with BMD. The observed positive correlations between adiposity markers with BMD suggest that higher hormone levels resulting from greater adipose tissue, rather than mechanical weight bearing, may activate bone remodeling in the SCI population. Further understanding of the relationships between adiposity and bone health may help to identify the effects of the drastic body composition changes during the acute and chronic phases of SCI. With increasing rates of SCI predicted in the coming decades (Noonan et al., 2012), research on accurate prevention, treatment, and rehabilitation of SLOP is necessary to improve the quality of life of individuals with SCI and reduce healthcare costs.

## Funding

Funding for this study was provided by the Canadian Institutes of Health Research, the Ontario Neurotrauma Foundation (2011-ONF-RHI-MT-888) and the Natural Sciences and Engineering Research Council (RGPIN 238819-13).

## Figures and Tables

**Table 1:** Participant and injury characteristics of SCI cohort. Values are mean (SD).* n = 29

Subject characteristics (n = 34)	
Male/female (n)	32/2
Age (years)	40.0 (10.9)
Height (m)	1.75 (0.08)
Mass (kg)	82.1 (16.6)
BMI (kg/m^2^)	26.5 (4.7)
WC (cm)	93.0 (13.6)
Body fat (%)*	28.4 (7.3)

Injury characteristics	# Participants

Motor complete/Incomplete (n)	17/17
Tetraplegia/Paraplegia (n)	22/12
Traumatic/Non-traumatic (n)	27/7
Time post injury (years)	12.7 (9.9)

**Table 2:** Outcome measures for participants with spinal cord injury (SCI) for whole group and for SCI compared to matched non-SCI comparison group.

		SCI	SCI vs non-SCI		p-Value
*Bone mineral density*					
Femoral neck BMD (g/cm^2^)	N	28	7	8	
	Mean (SD)	0.69 (0.19)	0.63 (0.16)	0.92 (0.12)	0.001
	T-score	− 1.04 (n = 5)	− 2.50 (n = 1)	− 0.9 (n = 2)	N/A
	Z-score	− 1.49 (n = 23)	− 1.70 (n = 6)	0.68 (n = 6)	N/A
Lumbar spine BMD (g/cm^2^)	N	27	8	8	
	Mean (SD)	1.12 (0.17)	1.12 (0.2)	1.08 (0.10)	0.630
	T-score	0.37 (n = 6)	0.15 (n = 2)	− 0.4 (n = 2)	N/A
	Z-score	0.29 (n = 21)	0.25 (n = 6)	0.07 (n = 6)	0.837
Visceral adipose tissue (kg)	N	29	8	8	
	Mean (SD)	3.11 (1.47)	2.75 (1.42)	2.29 (1.44)	0.533
	VAT (%)	29.1 (8.73)	26.7 (8.69)	22.0 (8.8)	0.298

**Total nutrient intake**	N	32	8	8	
Vitamin D (IU/day)	Mean (SD)	1224 (1315)	1321 (696)	396 (288)	0.007
	Range	155–7413	250–2246	4–907	
	% below EAR	31.3	25.0	62.5	0.149
Calcium (mg/day)	Mean (SD)	1611 (787)	1765 (679)	1613 (907)	0.712
	Range	63–3646	1014–2440	236–3117	
	% below EAR	9.4	0	25.0	0.149
Vitamin K (μg/day)	Mean (SD)	193 (212)	172 (175)	254 (289)	0.505
	Range	15–1181	17–528	66–934	
Protein (g/day)	Mean (SD)	82 (36)	90 (29)	93 (39)	0.859
	Range	23–186	51–139	12–144	
	% below EAR	25.0	12.5	12.5	1.000

**Blood biomarkers**	N	33	8	8	
25 (OH)D (nmol/L)	Mean (SD)	69.3 (23.3)	89.9 (14.3)	76.5 (19.8)	0.144
	Range	18–120	69–104	50–115	
Leptin (ng/mL)	Mean (SD)	14.8 (31.4)	5.1 (3.3)	5.7 (5.2)	0.770
	Range	0.21–180	0.21–10.6	1–16	
Insulin (pg/L)	Mean (SD)	288 (234)	204 (89)	236 (116)	0.543
	Range	58–1180	90–320	111–437	
Adiponectin (ng/mL)	Mean (SD)	40.5 (44.0)	24.9 (23.3)	18.7 (10.5)	0.505
	Range	8-200	11–81	7.2–39.9	

Values are mean (SD). BMD T-score was calculated in participants > 50 years; Z-score was calculated in participants < 50 years. p-Value represents independent *t*-test between matched SCI and non-SCI groups.

**Table 3:** Multiple linear regression analyses for measurements in participants with SCI (n = 25-27).

Variables	Unstandardized coefficients		r	P-value	95.0% CI
	B	Std. Error			
*BMD FN*					
Calcium intake	0.000	0.000	0.115	0.561	0.00–0.00
25(OH)D	− 0.002	0.002	− .221	0.268	0.00–0.00
Leptin	0.010	0.003	.529	0.005⁎	0.00–0.02
Insulin	0.000	0.000	.544	0.003⁎	0.00–0.00
Adiponectin	0.000	0.001	.227	0.255	0.00–0.00
VAT %	0.010	0.004	.444	0.018⁎	0.00–0.02

*BMD LS*					
Calcium intake	0.000	0.000	.026	0.899	0.00–0.00
25(OH)D	0.000	0.001	.009	0.996	0.00–0.00
Leptin	0.007	0.003	.392	0.048⁎	0.00–0.01
Insulin	0.000	0.000	.388	0.050⁎	0.00–0.00
Adiponectin	0.002	0.001	.429	0.029⁎	0.00–0.00
VAT %	0.008	0.004	.381	0.048⁎	0.00–0.02

⁎ P-value < 0.05.
